# Using machine learning to impact on long-term clinical care: principles, challenges, and practicalities

**DOI:** 10.1038/s41390-022-02194-6

**Published:** 2022-07-29

**Authors:** James J. Ashton, Aneurin Young, Mark J. Johnson, R. Mark Beattie

**Affiliations:** 1 Department of Paediatric Gastroenterology, Southampton Children’s Hospital, Southampton, UK; 2 Human Genetics and Genomic Medicine, University of Southampton, Southampton, UK; 3 Department of Neonatal Medicine, Princess Anne Hospital, University Hospital Southampton NHS Foundation Trust, Southampton, UK; 4 National Institute for Health Research, Southampton Biomedical Research Centre, University Hospital Southampton NHS Foundation Trust and University of Southampton, Southampton, UK

## Abstract

**Abstract:**

The rise of machine learning in healthcare has significant implications for paediatrics. Long-term conditions with significant disease heterogeneity comprise large portions of the routine work performed by paediatricians. Improving outcomes through discovery of disease and treatment prediction models, alongside novel subgroup clustering of patients, are some of the areas in which machine learning holds significant promise. While artificial intelligence has percolated into routine use in our day to day lives through advertising algorithms, song or movie selections and sifting of spam emails, the ability of machine learning to utilise highly complex and dimensional data has not yet reached its full potential in healthcare. In this review article, we discuss some of the foundations of machine learning, including some of the basic algorithms. We emphasise the importance of correct utilisation of machine learning, including adequate data preparation and external validation. Using nutrition in preterm infants and paediatric inflammatory bowel disease as examples, we discuss the evidence and potential utility of machine learning in paediatrics. Finally, we review some of the future applications, alongside challenges and ethical considerations related to application of artificial intelligence.

**Impact:**

- Machine learning is a widely used term; however, understanding of the process and application to healthcare is lacking.
- This article uses clinical examples to explore complex machine learning terms and algorithms.
- We discuss limitations and potential future applications within paediatrics and neonatal medicine.

## Introduction

The rise of artificial intelligence (AI) and machine learning (ML) as a routine tool for business, government, and institutions, to better understand huge volumes of data has also opened doors for utilisation in healthcare. ‘Big data’ is not a novel concept in medical research or clinical care, however the ability to process and understand highly dimensional and longitudinal information for clinical benefit, is now far more attainable. This review will discuss the principles underlying ML and how these have been, and can be, applied to long-term clinical care for the benefit of patients, illustrating this with the disease models of preterm nutrition and paediatric inflammatory bowel disease (IBD).

### What is machine learning?

**Fig. 1:**
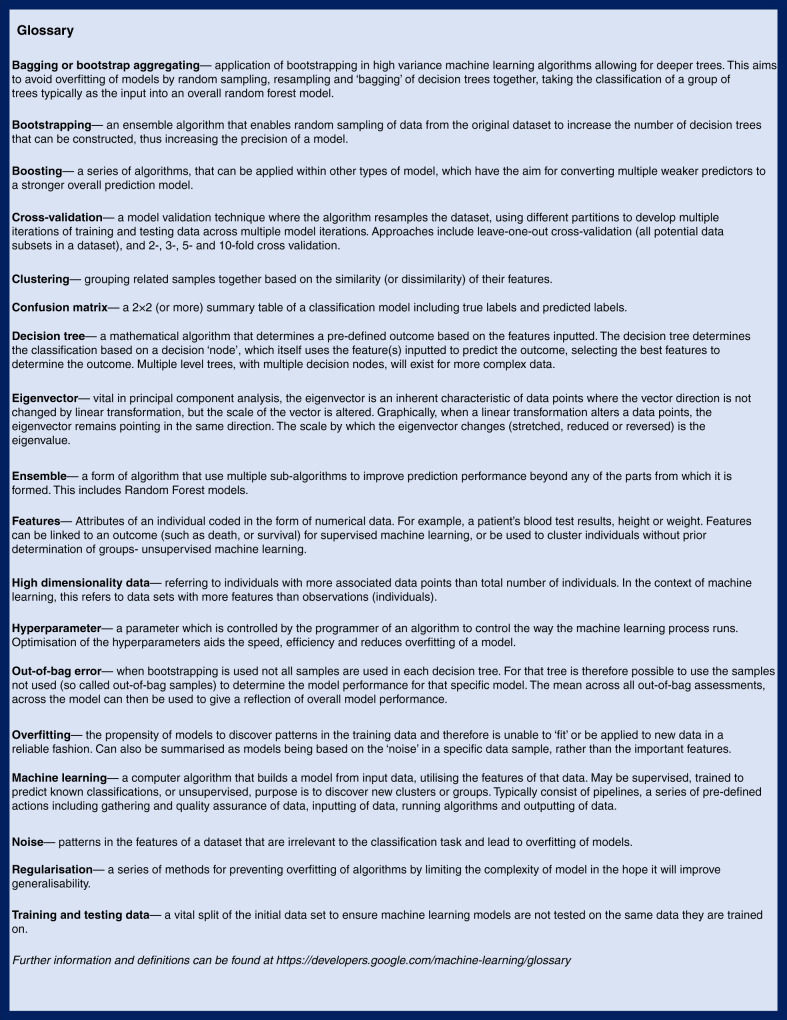
Glossary of terms related to machine learning and machine learning algorithms.

ML is a subclassification of AI, consisting of a predefined algorithm able to use statistical models to learn and discover patterns in data.^1^ ML can be broadly divided into supervised ML, models that are instructed to discover patterns related to specific predefined groups in order to classify individuals, and unsupervised ML, where algorithms discover groups based on the data without prior instruction. An example of supervised ML in the context of clinical care would be developing a diagnostic classification tool for inflammatory bowel disease, classifying patients as ulcerative colitis or Crohn’s disease based on their distribution of inflammation at diagnosis.^2^ Unsupervised models look for (new) groups, with an example being clustering of patients with cystic fibrosis by underlying clinical variables with the objective of discovering groups associated with worse or better outcomes.^3^ Important terms have been defined in Fig. 1.

### An overview of machine learning models

**Fig. 2:**
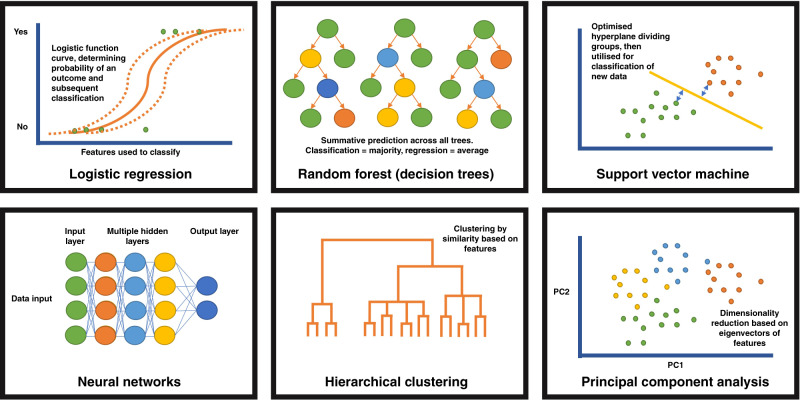
Visual representation of the more commonly used machine learning algorithms discussed within this article.

There are numerous mathematical models that may be considered machine learning. The application of these in the correct context and with high-quality data is vital to achieving interpretable outcomes. The models are reflected visually in Fig. 2.

#### Logistic regression

Machine learning incorporates regression analysis into many different more complex models. Regression utilises features (typically termed independent variables) to predict the outcome (dependant variable). Logistic regression is, in essence, a classification algorithm which uses the logistic function and fitted model parameters (including weighting) to determine whether features are able to classify the binary (0 or 1) outcome correctly. A potential modified use of logistic regression, used in big population data sets, is propensity score matching. Here, observational studies comparing a group (or groups) to a reference population, are adjusted for potential confounders leading to specific individuals being more likely to fall within a specific group (e.g. receive a certain treatment) based on measurable characteristics. Comparison can then occur between individuals within the same propensity score bracket to ensure a fairer retrospective comparison.

#### Random forest and decision trees

These are a well-established classification or regression model. Random forest models consist of multiple decision ‘trees’, these trees solve problems through splitting data based on inputted features, for example a tree might use demographic data to predict a specific outcome (such as risk of developing a disease), with the tree outputting based on the data splits occurring within that tree. For example, the splits may follow that patients who are male, aged >15 years, Caucasian = have disease, which is that tree’s output. Other decision trees will reach different predictions due to different data splits (routes taken through the tree). The outcome selected by the majority of the decision trees is used in classification, whereas the average across all trees is used in a regression model. The ‘forest’ element involves application of multiple decision trees with bagging (or bootstrapping) of the sample (and potentially the features), this helps to avoid ‘overfitting’ of the model to the data where individual trees find patterns in the data which are not relevant to the outcome. It is possible to extract feature importance from models through determining the how each feature performs at splitting the data through a number of mathematical methods.

#### Support vector machines

This well-established form of supervised machine learning able to classify and regress against outcome data. Typically applied as binary classifier although may be applied to multiple outcomes with modification. The algorithm creates a series of ‘hyperplanes’, which are decision boundaries between datapoints. The number of features within the model determine the dimensionality in which the hyperplane is drawn (2 points = a line, 3 points = plane, >4 points = mathematical but unable to image). The hyperplane furthest from the different outcome datapoints, i.e. separating the outcomes best based on their features is the ‘decision boundary’. This is boundary is then applied to testing data to classify into groups.

#### Neural networks

This model is based on the functioning of human brains. A neural network consists of connected nodes, termed ‘neurons’. Each node is connected to other nodes, the inputs for the initial layer of nodes are features (numerical values from the input data), with subsequent layers receiving the outputs of other nodes in the previous layer. Each node has a single output, which is passed to downstream nodes. The final output nodes complete the task (typically classification but can be an unsupervised model), with data potentially passing through many layers of nodes to reach this point.

Each decision node can be considered like a linear regression model with input features and weighting of feature importance related to the outcome. The output of a node is determined by a threshold value, if exceeded the node will output data, ‘fire’, to the subsequent layer of nodes or complete the task.

The term deep learning refers to neural networks with >3 layers of nodes, with the same algorithms as for more superficial neural networks, but with more decision points.

#### Clustering (hierarchical, *k*-means etc.)

Clustering of data can be considered a basic form of unsupervised machine learning. It is a mathematical way of assessing similarity of data, of which there are a number of methodologies. *K*-means clustering is a commonly applied tool. Here the number of groups are specified and by calculating iterations of the distance of data points from the centre of randomly calculated groups of data, points are clustered to specific groups. The distance between points is determined by the features of that point.

Hierarchical clustering may refer to bottom–up (agglomerative, starting with all individual data points and merging), or top–down (divisive, starting with a single cluster and dividing) clustering. The distance between points is determined by the features of that point, and the closer to one another they are, the smaller cluster they form. Visualisation of hierarchical clustering is typically using a dendrogram, here each individual point is represented and connected by lines to all other points. The distance between points reflects how similar, or dissimilar a point is to another point, or a cluster is to another cluster. There are a number of ways of visualising the distance between clusters including maximal, minimal and average dissimilarity of points (within the cluster).

#### Dimensionality reduction (principal component analysis (PCA))

Perhaps not considered a true form of machine learning, dimensionality reduction algorithms aim to aid visualisation and understanding of highly dimensional (complex with many features) data. It can also be used to explore complex data sets and make predictions related to clustered individuals. PCA is the best-known method, here the algorithm reduces the data to the minimal complexity that explains the maximal variance in the data set. The principal components are the eigenvectors of any feature matrix, and data sets can be summarised by any number of eigenvectors. Data can then be visualised in 2D (two principal components) and 3D (3 principal components) representations.

## The importance of data processing and quality control

Often machine learning models are seen as ‘black boxes’ for data processing, with little consideration for utilisation of the correct model, high quality input data and correct interpretation. However, it is vital to appreciate that the term ‘garbage in, garbage out’ is highly appropriate when using machine learning. The processing and application of machine learning models to clinical data has some key differences to application of classic statistical or epidemiological approaches. Ensuring that data sets are balanced when considering cases and controls (in training and testing data), and the models are optimised allows for improved generalisability and widespread application.

The primary consideration before applying data to a machine learning strategy is the appropriateness of the model, and whether conventional statistics would be appropriate. Choosing the correct type of algorithm for a task is paramount to interpretable, and useful results. The second consideration is the quality of the data, including consideration of missing data. Typically, data must be numerical (excluding image and text recognition). High-quality features relevant to the outcome and potentially selected based on a priori knowledge, are likely to result in improved model performance. Splitting data into training and testing sets, with balanced outcomes between sets, is vital. Strategies can be employed for unbalanced data sets, such as resampling. Next, optimisation of the model through assigning values to the potential variables within the algorithm (bagging strategies, pruning, classification vs regression etc.) alongside hyperparameter tuning to optimise speed, efficiency and performance of the model, while minimising overfitting. The importance of optimisation and avoiding overfitting is paramount to the generalisability of models. In more simple terms, repeated sampling and resampling of the data (bootstrapping) to maximise the ability of the model to learn must be balanced against the model learning unimportant patterns in the data (overfitting), which prevent generalisability to the testing data.

Finally, once the model has run and optimisation has occurred, there is the importance of interpretation within the context of the research question. Asking a focused question at the start of the process vastly improves the interpretability, and potential clinical application, of any results.

Metrics associated with model performance on the testing data are also important to understand. These include relatively simple assessment of performance such as relative feature importance, precision and recall (can be visualised as a precision-recall curve and interpretated like a receiver operator curve) and the F1 statistic (combination of precision and recall). Certain models, such as random forest, will produce a confusion matrix which allow visualisation of the performance when comparing the predicated classification (from the model) versus the true classification.

Application of the model to external and large data sets ensures reproducibility beyond the training and testing conditions within the original algorithm.

## Preterm nutrition—using clinical data to optimise growth

### The challenge of nutrition for preterm infants

Preterm infants are prone to poor growth. Despite international recommendations that their growth should mimic that of the equivalent foetus in utero, they frequently fail to achieve this pace of growth.^4,5^ The consequences of this shortfall are likely to be important, with better growth in early life associated with improvements in neurodevelopmental outcome.^6,7^ The growth of the preterm infant is influenced by numerous factors.^8^ Most obviously, nutritional intake is bound to be influential. However, growth is also likely to be influenced by a range of other clinical features, including antenatal factors, complications of prematurity (such as bronchopulmonary dysplasia), management strategies (especially the use of corticosteroids) and pro-inflammatory states caused by infection. Recent developments in multi-omics approaches are also beginning to elucidate genomic, metabolomic and microbiome influences on growth. These influences combine to make a complex landscape, and it can be difficult to navigate these diverse factors when looking for insights into modifiable targets for improving growth. Machine learning approaches are likely to provide powerful tools for understanding such complex observational data.

### Data on the neonatal intensive care unit

Despite the challenges of understanding such a complex field, the neonatal intensive care unit provides a wealth of opportunities to gather high quality data. During the course of their clinical care, preterm infants are subjected to intensive monitoring of their physiological state and biochemical parameters. Furthermore, detailed data are routinely gathered to describe demographic information and their antenatal exposures. Intakes of nutritional products are carefully monitored. In the UK, demographic details, antenatal problems, summarised daily management and clinical outcomes are gathered on the National Neonatal Research Database (NNRD).^9^ These data undergo quality control but the reliability of data on the database is variable, with significant discrepancies in key outcomes (including bronchopulmonary dysplasia and neurodevelopmental progress) and in aspects of clinical care, such as the type of milk feeds provided.^10,11^ More detailed data, including nutritional intake and minute-to-minute physiological observations have generally been recorded on paper charts, making it laborious to collate such data into a form appropriate for statistical analysis. However, there has been a recent increase in the use of comprehensive electronic clinical information systems in the neonatal intensive care unit.^12^ These systems can automatically extract and record observations from monitors and equipment such as ventilators, although efforts to unify recording of data across different clinical information systems are in a nascent stage.^13^ They can also be used to store clinical data (such as nutritional product intake) in a structured format, rendering the resulting data much more accessible to statistical analysis. Simultaneously, genomic, metabolomic and microbiome methods have become cheaper and more accessible. The facilities and expertise to store and analyse the resulting data are also becoming more available. Taken together, these developments provide an exciting opportunity to gain insights into the growth of the preterm infants, but such complex data will require advanced statistical techniques to draw out meaning.

### Progress with machine learning

To date, there have been limited attempts to use machine learning to understand the nutrition and growth of preterm infants. A structured search of MEDLINE identified eight papers using machine learning techniques to investigate elements of the growth and nutrition of preterm infants in the past 10 years, Table 1. It is notable that six of the eight were published in 2019 or later, suggesting that interest in machine learning approaches to these data has increased in the last three years.

**Table 1:** Papers identified by a structured search of MEDLINE using machine learning techniques to investigate the nutritional and growth of preterm infants in the past 10 years.

Data type	Authors	Title	Year	Journal	Main topic of focus	Type of statistical technique
Clinical	Porcelli et al.^32^	Comparison of new modelling methods for postnatal weight in ELBW infants using prenatal and postnatal data	2015	J Pediatr Gastroenterol Nutr	Modelling influences on early weight gain	Neural networks
Clinical	Irles et al.^33^	Estimation of neonatal intestinal perforation associated with necrotising enterocolitis by machine learning reveals new key factors	2019	Int J Environ Res Public Health	Risk of intestinal perforation	Neural networks
Clinical	Fu et al.^34^	Integration of an interpretable machine learning algorithm to identify early life risk factors of childhood obesity among preterm infants: a prospective birth cohort	2020	BMC Medicine	Development of obesity in ex-preterm infants	Gradient-boosted trees
Clinical	Wong et al.^35^	Predicting protein and fat content in human donor milk using machine learning	2021	J Nutr	Modelling influences on donor breastmilk composition	Random forest; Gradient-boosted trees
Clinical/microbiome	Lugo-Martinez et al.^14^	Integrating longitudinal clinical and microbiome data to predict growth faltering in preterm infants	2022	J Biomed Inform	Clinical and microbiomic influences on growth faltering	Random forest; Hidden Markov
Metabolomics	Wilcock et al.^36^	The metabolomics of necrotising enterocolitis in preterm babies: an exploratory study	2016	J Matern Fetal Neonatal Med	Necrotising enterocolitis	Principal component analysis
Metabolomics	Younge et al.^37^	Disrupted maturation of the microbiota and metabolome among extremely preterm infants with postnatal growth failure	2019	Sci Rep	Growth failure	Partitioning around medoid (PAM) clustering
Service delivery	Greenbury et al.^38^	Identification of variation in nutritional practice in neonatal units in England and association with clinical outcomes using agnostic machine learning	2021	Sci Rep	Defining clusters of practice	Unsupervised clustering

Papers identified by a MEDLINE search: (“preterm” OR “premature” OR “neonatal” OR “newborn”) AND (nutrition OR growth) AND (“big data” OR “artificial intelligence” OR “machine learning” OR “modeling” OR “modelling”) AND “last 10 years”[dp]. Papers identified by search: 375. Papers selected after screening of titles and abstracts: 8.

These papers address a wide range of questions. Five focused on clinical problems, investigating general influences on growth or the risk of specific complications. One of these papers addressed the composition of donated breastmilk. Three papers focused on the microbiome or metabolome and used cluster identification techniques to discover patterns in the data. The eighth paper also used a clustering technique, in this case to explore variations in clinical practice and the influence of these variations on clinical outcomes.

A recent paper by Lugo-Martinez and co-workers provides the most comprehensive example of the potential for machine learning techniques to provide insights into the multiple influences on the growth of the preterm infant.^14^ They first assessed models using clinical data to predict growth failure, and it is interesting to note that straightforward logistic regression provided superior models to a random forest approach in this case. When microbiome data were included, a hidden Markov model outperformed those based on logistic regression alone.

This paper, along with the more focused findings of the other identified studies, highlights the potential for machine learning approaches to inform investigation into the growth and nutrition of preterm infants, especially when used in the context of emerging data acquisition, data storage and multi-omics methods.

## Paediatric inflammatory bowel disease—using ‘big data’ for prediction and personalised therapy

Paediatric IBD has seen a wealth of machine learning effort. The major challenge in IBD is the heterogeneity of disease, making predicting complications, disease behaviour and response to therapy in individual patients, highly complex.^15^ Tools to aid making a precise diagnosis leading to personalisation of management are highly desirable, with machine learning frequently employed in an effort to understand highly dimensional molecular, biochemical and clinical data.

A recent systematic review identified numerous articles pertaining to machine learning and IBD.^16^ While the minority focus on paediatric patients, the number of studies is expanding year on year. Here we focus on three key areas utilising machine learning, classification of disease (and developing new patient groups), prediction of outcomes and prediction of response to therapy.

### Disease classification and precision diagnosis

Perhaps the vanguard of machine learning in paediatric IBD is to aid with disease classification. The potential of this strategy is two-fold, first to classify patients into known disease categories (supervised classification) such as Crohn’s disease and ulcerative colitis. Secondly, there is the opportunity to develop novel patient groups, where clusters are formed based on the underlying clinical or molecular features, rather than overall phenotype.

Mossotto et al. describe a machine learning method using a support vector machine with recursive feature elimination to classify patients into Crohn’s disease and ulcerative colitis diagnostic subgroups based on endoscopic and histological findings.^2^ The model was able to classify patients to the clinician assigned diagnostic subgroup with an accuracy of 82.7% utilising 8 features (from a possible 20 features). Within the manuscript the authors also perform unsupervised machine learning, in the form of a hierarchical clustering and PCA. This identified four subgroups of IBD, with groups 3 and 4 enriched for ulcerative colitis and Crohn’s disease, respectively. A further clustering experiment from the same group identified novel groupings of patients at the point of diagnosis based only on blood test results. Ashton et al. demonstrated 12 patient clusters based on C-reactive protein (CRP), erythrocyte sedimentation rate, white cell count, haemoglobin, platelet count, packed cell volume, albumin and alanine transferase results.^17^ Within this, two outlying groups were enriched for Crohn’s disease (high CRP and low albumin) and ulcerative colitis (normal albumin and low haemoglobin). A further novel cluster of patients was characterised by isolated increase in white cell count, with normal other bloods.

Dhaliwal et al. utilised a random forest classifier to attempt to create a model able to distinguish between colonic Crohn’s disease and ulcerative colitis.^18^ Their model performed with excellent accuracy on a testing set (100%) and was able to identify 7 features from a possible 28 clinical, histological, endoscopic and radiological features, important in classification of colonic disease.

Considering novel molecular diagnoses, Ashton et al. utilised whole-exome sequencing and targeted RNA sequencing of paediatric IBD patients to determine the impact of variation within the NOD-signalling pathway.^19^ Within these analyses they performed a principal component and hierarchical clustering experiment, identifying specific groups of patients where perturbation in genes, or gene complexes, translated to clustering based on RNA expression. These clusters have the potential to represent precise molecular diagnostic groups, and in turn enable targeted therapy based on the underlying genetic defect.

## Outcome prediction

IBD outcome prediction using machine learning is the closest to clinical application. A number of large prospective cohort studies have identified predictive clinical, molecular and biochemical features, associated with complex disease and disease progression. Kugathasan et al.^20^ used an inception cohort of over 900 Crohn’s disease patients to predict which patients would get complications (penetrating or stricturing disease). Utilising a competing risk model, a form of conventional statistics accounting for multiple survival outcomes (in this case inflammatory, penetrating or stricturing disease) but incorporating a number of clinical and molecular features that were reduced to principal components including ileal gene expression and genetic risk scores. The overall model performed with an area under the curve (AUC) of 0.7 and included age, ethnicity, disease location and serological features. Hyams et al.^21^ performed a similar analysis using a logistic regression to predict features associated with corticosteroid-free remission in patients with ulcerative colitis. The team reported a predictive model with an AUC of 0.75 including clinical (most notably remission by week 4) and molecular features. Specifically, an antimicrobial colonic gene signature and presence of *Sutterella* bacteria were associated with lower rates of steroid-free remission at 1 year.

Ungaro et al. adopted a different strategy in patients from the same cohort used by Kugathasan and colleagues. Here they employed a random survival forest model (with out-of-bag error performance assessment) to predict penetrating or stricturing disease complication in 265 Crohn’s disease patients using 92 inflammatory proteins from blood plasma.^22^ Considering penetrating disease, five protein markers predicted development with an AUC of 0.79, compared an AUC 0.74 for clinical variables only. Four proteins predicted stricturing disease with an AUC of 0.68, with clinical features being no better than chance (AUC 0.52).

### Prediction of response to therapy

A further avenue for machine learning application in IBD is to determine the response of patients to specific therapies. It is established that even high cost biologics have primary non-responder rates that can exceed 50%.^23^ Douglas et al. used a random forest classifier and microbial sequencing data to determine the response to induction therapy.^24^ They conducted random forest classifiers for treatment response on a number of independent data sets, and then combined the top features from these into a summative model. Within this the authors report an accuracy of 94.4% in prediction of response to induction therapy, identifying specific bacterial taxa and KEGG-pathways predicting outcomes. Jones and colleagues performed a similar experiment, predicting response to exclusive enteral nutrition in Crohn’s disease patients using bacterial function and composition.^25^ Again, a random forest classifier was employed with a leave-one-out cross-validation. When including clinical features (disease location and behaviour), alongside bacterial species abundance and richness, the AUC was reported to be 0.90.

### Future applications in IBD

Increasingly there is interest in the use of machine learning for other applications in IBD. While these are largely in their infancy for paediatric disease, the use of AI for endoscopy and histological interpretation, home monitoring apps and drug design all show promise and remain attractive avenues for research and investment.^1^ The role of machine learning to aid clinicians with diagnostics, treatment stratification and improving outcomes for patients must be at the centre of further implementation (Table 2).

**Table 2:** Selected papers identified through a structured MEDLINE search.

Data type	Authors	Title	Year	Journal	Main topic of focus	Type of statistical technique
Clinical	Mossotto et al.^2^	Classification of paediatric inflammatory bowel disease using machine learning	2017	Sci Rep	Diagnostic classification and cluster discovery	Support vector machine, hierarchical clustering
Clinical	Ashton et al.^17^	Analysis and hierarchical clustering of blood results before diagnosis in paediatric inflammatory bowel disease	2020	IBD	Cluster discovery using clinical data	Hierarchical clustering
Exome and transcriptome	Ashton et al.^19^	Deleterious genetic variation across the NOD signalling pathway is associated with reduced NFKB signalling transcription and upregulation of alternative inflammatory transcripts in paediatric inflammatory bowel disease	2022	IBD	Cluster discovery using molecular data	Hierarchical clustering
Clinical	Dhaliwal et al.^18^	Accurate classification of paediatric colonic inflammatory bowel disease subtype using a random forest machine learning classifier	2021	JPGN	Diagnostic classification	Random forest classifier
Clinical, molecular + biochemical	Kugathasan et al.^20^	Prediction of complicated disease course for children newly diagnosed with Crohn’s disease: a multicentre inception cohort study	2017	The Lancet	Prediction of complex disease	Competing risk model
Clinical, molecular + biochemical	Hyams et al.^21^	Clinical and biological predictors of response to standardised paediatric colitis therapy (PROTECT): a multicentre inception cohort study	2019	The Lancet	Prediction of corticosteroid-free remission	Logistic regression
Biochemical	Ungaro et al.^22^	Machine learning identifies novel blood protein predictors of penetrating and stricturing complications in newly diagnosed paediatric Crohn’s disease	2021	APT	Prediction of complex disease	Random forest Survival
Molecular	Douglas et al.^24^	Multi-omics differentially classify disease state and treatment outcome in paediatric Crohn’s disease	2018	Microbiome	Prediction of response to induction treatment	Random forest classifier
Molecular	Jones et al.^25^	Bacterial taxa and functions are predictive of sustained remission following exclusive enteral nutrition in paediatric Crohn’s disease	2020	IBD	Prediction of response to exclusive enteral nutrition	Random forest classifier

All studies use machine learning techniques to investigate the classification, group discovery, outcome and therapy prediction in paediatric inflammatory bowel disease.

## Machine learning—potential uses and future challenges

When considering the application of machine learning techniques, the question to be answered is all important. Machine learning is not a panacea for the limitations of conventional statistics, nor will machine learning methodologies magically discover perfectly accurate disease prediction models. However, machine learning, as a concept, does hold great promise and can complement conventional statistics and study design. The utilisation of machine learning in highly dimensional data coupled with vast patient numbers can produce excellent results with real impact for patients in areas such as image interpretation and drug discovery.^26,27^ Acknowledgement of the challenges of frequent lack of reproducibly and external validation, and moving towards transparency, standardised reporting and improved understanding of machine learning research, is important to take the next steps towards clinical impact.^28^

### Utilisation of real-world data

A major challenge of modern healthcare relates to the vast quantities of data collected on each patient. This is compounded in specific care settings such as intensive care, where electronic patient records are able to collect and store constant physiological measurements throughout the patient stay. This challenge also presents an opportunity for utilisation of machine learning for real-world data, collated as a routine part of the patient visit. Beyond this, using tools such as natural language processing and image interpretation (through neural networks) may allow for older records to be incorporated into future research. Investment in improvement of prospective data collection, creation of resilient and robust databases, and improving overall data quality will translate to future clinically useful application of machine learning.

### Ethical implications

In an era of big data, there are clear ethical considerations relating to the use of patient information in research. With machine learning generally models benefitting from larger data sets the inclusion of anonymised data is frequent. There has been recent controversy around sharing of data sets with tech-firms such as Google, with millions of individuals having unconsented records released from the national health service in the UK, largely for the purpose of development of predictive machine learning models.^29^ More widely, prediction models in paediatric disease frequently utilise molecular data, with the use of genomics likely to dramatically increase over the next 5–10 years. Ethical implications of broad consent for use of genomic data has been highlighted by the 100,000 genomes project in the United Kingdom. Where patients are broadly consented for research focused on their disease, complex decisions about feedback of unexpected findings, uncertain discoveries and unrelated disease must be case by case and revisited with changing societal acceptance.^30^ In the context of machine learning approaches to genomic data this creates even greater challenges as the drivers of prediction models are not always clear.^31^

### Potential applications within paediatrics

Numerous diseases of childhood may benefit from machine learning, beyond the examples given in this article. Chronic, complex conditions with high intra-disease heterogeneity are potential low hanging fruit for prediction modelling. This group may include many rheumatological and dermatological disorders where choice of, and response to, immunosuppressive therapy is important.

Pairing highly dimensional data such as continuous observations collected during intensive care stays, with outcomes, admission durations and complications, may be useful in highlighting patients at an early stage who require escalation of care, or who can be discharged to the ward earlier.

Even common seasonal childhood illness such as bronchiolitis, viral induced wheeze and gastroenteritis may benefit from machine learning research. Utilising clinical parameters and discharge outcomes may help to identify factors associated with morbidity or patients who can be safely discharged.

**Fig. 3:**
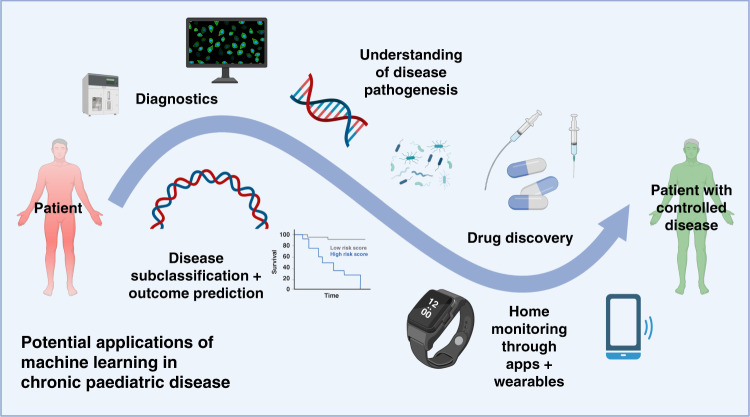
Summary visualisation of five potential application for machine learning strategies in paediatric chronic disease. This includes utilisation in (1) diagnostics, such as image or result interpretation, (2) disease outcome and prediction, such as novel groups associated with complications, (3) improved understanding of pathogenesis, such as interpretation of genomic or microbiome data, (4) drug discovery, including repurposing medication, and (5) home monitoring, specifically through use of data from applications and wearables. Created with BioRender.com.

Figure 3 summarises some of the potential applications of machine learning for chronic disease, including the opportunity to improve diagnostics and integration of data from applications or wearable technology.

### Why machine learning is not always the answer

It is important to recognise that machine learning strategies are not always the answer. Conventional statistics continue to play an important role, especially in time-series and clinical trial data. Additionally, there must remain human oversight in key areas such as prescriptions, clinical advice and image reporting, although ML is likely to increasingly become an important adjunct in healthcare delivery. The reproducibility and generalisability of ML models is a frequent criticism of research. Increasing the participation of less represented groups in the training of models, applying models to external data sets and moving towards a standard of reporting are all key steps in allowing ML to be used well in healthcare. This is particularly pertinent in paediatrics, where children’s data is often not represented in large ML research to date.

A final consideration is how poorly understood ML and AI concepts are in the general medical field. Improving training to aid interpretation of research and implementation into clinical practice is of the utmost importance.

## Conclusions

In this review, we discuss the potential of ML to aid with management of long-term paediatric conditions, including some of the basic approaches. We utilise two clinical problems, preterm nutrition and IBD, to demonstrate the potential of ML to improve patient care in the future. Finally, we discuss the framework for implementation, including opportunities and challenges for translational research. ML has huge potential but must be used correctly, it is likely that the next 5–10 years will see routine clinical ML tools emerge to act as high sophisticated adjuncts to benefit patient care.

## Data Availability

No new data are included in this review article.
